# Construction and use of a *Cupriavidus necator* H16 soluble hydrogenase promoter (P_SH_) fusion to * gfp* (green fluorescent protein)

**DOI:** 10.7717/peerj.2269

**Published:** 2016-07-26

**Authors:** Bat-Erdene Jugder, Jeffrey Welch, Nady Braidy, Christopher P. Marquis

**Affiliations:** 1 School of Biotechnology and Biomolecular Sciences, University of New South Wales,Sydney,NSW,Australia; 2 Centre for Health Brain Ageing, School of Psychiatry, University of New South Wales,Sydney,NSW,Australia

**Keywords:** Soluble hydrogenase, *Ralstonia eutropha*, *Cupriavidus necator*, Green fluorescent protein, Promoter

## Abstract

Hydrogenases are metalloenzymes that reversibly catalyse the oxidation or production of molecular hydrogen (H_2_). Amongst a number of promising candidates for application in the oxidation of H_2_ is a soluble [Ni–Fe] uptake hydrogenase (SH) produced by *Cupriavidus necator* H16. In the present study, molecular characterisation of the SH operon, responsible for functional SH synthesis, was investigated by developing a green fluorescent protein (GFP) reporter system to characterise P_SH_ promoter activity using several gene cloning approaches. A P_SH_ promoter-gfp fusion was successfully constructed and inducible GFP expression driven by the P_SH_ promoter under de-repressing conditions in heterotrophic growth media was demonstrated in the recombinant *C. necator* H16 cells. Here we report the first successful fluorescent reporter system to study P_SH_ promoter activity in *C. necator* H16. The fusion construct allowed for the design of a simple screening assay to evaluate P_SH_ activity. Furthermore, the constructed reporter system can serve as a model to develop a rapid fluorescent based reporter for subsequent small-scale process optimisation experiments for SH expression.

## Introduction

Hydrogenases are ubiquitous enzymes with reversible hydrogen oxidation or production activity (Schlegel, Kaltwasser & Gottschalk, 1961; Friedrich, Friedrich & Bowien, 1981). A wide variety of H_2_ oxidizing organisms including aerobes, anaerobes, autolithotrophs, heterotrophs, fermentative, photosynthetic and thermophilic microorganisms, have been described and are capable of producing and utilising endogenous uptake (H_2_ oxidising) hydrogenases. [Ni–Fe] hydrogenases are the best studied class of the uptake hydrogenases, that are characterised by their H_2_ oxidation activity and tolerance to molecular O_2_. These hydrogenases consist of a large subunit hosting the [Ni–Fe] active site and a small subunit housing the Fe–S cluster (Vignais, Billoud & Meyer, 2001; Vignais & Colbeau, 2004). The Knallgas bacteria *C. necator* H16 (formerly *Ralstonia eutropha or Alcaligenes eutropha*) is a chemolitho-autotrophic proteobacterium that is capable of growing both autotrophically using molecular hydrogen as the sole energy source and heterotrophically using organic compounds as the energy source (Pohlmann et al., 2006). *C. necator* H16 hosts three distinct O_2_-tolerant hydrogenases (Burgdorf et al., 2005); a membrane-bound hydrogenase (MBH), a soluble hydrogenase (SH) and a regulatory hydrogenase (RH). Under heterotrophic growth conditions, the expression of [Ni–Fe] uptake hydrogenases in *C. necator* H16 is induced on poorly utilised carbon sources (e.g., glycerol). Culture of this organism in minimal medium FGN (fructose-glycerol-nitrogen) is characterized by initial growth on the preferred fructose carbon source with hydrogenase expression repressed, followed by de-repression of hydrogenase expression as the organism switches to growth on the less-preferred substrate glycerol upon fructose exhaustion (Schlegel, Kaltwasser & Gottschalk, 1961; Friedrich, Friedrich & Bowien, 1981). In terms of potential application, oxygen-tolerant soluble hydrogenases (such as that produced by *C. necator*) show tremendous promise as bioelectrocatalysts in hydrogen fuel cells (Lamle, Albracht & Armstrong, 2004; Vincent et al., 2005; Jugder et al., 2013) and for mediating reduction reactions such as the reduction of NAD^+^ (Burgdorf et al., 2005).

The gene clusters for the three hydrogenases of *C. necator* H16 occupy a region of approximately 90 kbp of the megaplasmid pHG1. The SH is one of the most promising candidates for application in H_2_-based technologies owing to its H_2_ oxidation activity, oxygen tolerance, relatively favourable purification process and high expression under heterotrophic growth conditions (Burgdorf et al., 2005). The structural and accessory *hox* genes and maturation *hyp* genes of the SH reside on the large SH operon (10 kb) (Schwartz et al., 2003; Schwartz, 2009). A strong promoter, P_SH_, for these genes was identified in an upstream region of *hoxF* by primer extension analysis. The P_SH_ promoter is recognised by the sigma factor *σ*^54^ (RpoN) of the RNA polymerase and its sequence was proposed as 5′-TTGGCGCACATCCTGC-3′ (Schwartz, Gerischer & Friedrich, 1998). It has been well reported that one of two physiological conditions must be met to induce/de-repress the P_SH_ promoter and subsequently express the hydrogenase genes in *C. necator* H16. Under the first condition, H_2_ must be available in the growth media and typically a mixture of H_2_, CO_2_ and O_2_ with a volume ratio of 8:1:1 (autotrophic growth) has been employed widely to achieve induction in defined media. The second alternate inducing condition is achieved by the absence of preferentially utilized carbon and energy sources, such as fructose, in the medium which is conveniently achieved by using FGN medium whereby substrate shift occurs from fructose to glycerol under heterotrophic conditions (Friedrich, Friedrich & Bowien, 1981; Schlegel, Kaltwasser & Gottschalk, 1961; Jugder et al., 2015).

The use of reporter genes fused to a gene of interest has been widely reported for studying gene expression and promoter activity in a diverse array of living organisms. These reporters can be classified into conditional and non-conditional genes based on their need of an external substrate for detection (Xiong et al., 2012). Green fluorescent protein (*gfp)* has been amongst the most commonly used reporter genes since its first use as a reporter for gene expression in 1994 (Chalfie et al., 1994). As a reporter, *gfp* has great advantages over other reporters such as; direct real-time visualisation in living systems, little or no cytotoxicity on host cells, small size, and the availability of different mutants with modified spectral wavelengths (Xiong et al., 2012; Carroll & James, 2009). The use of *gfp* as a reporter gene was studied in *C. necator* cells with regard to polyhydroxyalkanoate (PHA) production (York et al., 2001; Fuchslin et al., 2003; Barnard et al., 2005). It has been reported that the expression of the *gfp* gene that is fused to the phaP promoter of phasin proteins, which are directly related to PHA synthesis, can be driven by the phaP promoter and thereby used as a tool to monitor PHA production. Nevertheless, P_SH_ promoter-driven GFP expression has not been reported in *C. necator*, to our knowledge.

We herein designed a recombinant reporter system to analyse the P_SH_ promoter activity in *C. necator* H16 utilising a mutant recombined operon comprising of a *gfp* gene assembled in a suicide vector, which is integrated within the megaplasmid pGH1. This tool allows for the analysis of potential growth conditions that de-repress promoter activity by monitoring the induction of the *gfp* gene expression. Employing qRT-PCR methodologies is time consuming and cannot effectively be applied to broad screening strategies to determine conditions associated with elevated SH production. The use of a simple visual reporter, such as GFP, would potentially be a time-saving and robust screening tool to investigate alternative growth conditions for potentially obtaining a higher yield of the SH from *C. necator* H16 by measuring GFP fluorescence emitted by the generated mutant strains. Increased SH specific productivity would also potentially improve recovery of active soluble hydrogenase.

## Materials and Methods

### Bacterial strains, growth conditions, plasmids and oligonucleotides

*C. necator* H16 (*Cupriavidus necator*, DSM 428) was routinely cultivated heterotrophically in minimal medium FGN as described in our previous work (Jugder et al., 2015; Jugder et al., 2016). The transconjugants were also grown under a hydrogenase repressing condition in FN medium (FGN medium without glycerol) and hydrogenase de-repressing condition in GN medium (FGN medium without fructose). *E. coli* strains were grown in Luria–Bertani medium (LB) except for conjugation processes where low-salt LB supplemented with 5% sucrose was used. *E. coli* DH10B containing the pJQ200mp18 suicide vector (ATCC 77485) was cultivated on LB media supplemented with gentamicin (15 μg/mL). *E. coli* S17-1 was maintained in LB media supplemented with trimethoprim (10 μg/mL). For blue/white screening, 100 μg/mL of ampicillin, 80 μg/mL of *X*-gal (5-bromo-4-chloro-3-indolyl-*β*-d-galactopyranoside) and 0.5 mM IPTG (isopropylthio-*β*-galactoside) were added to the LB agar media. SOC medium was used for transformation of *E. coli* JM109 High Efficiency Competent Cells. *C. necator* and *E. coli* strains were cultivated at 30 and 37 °C, respectively. The strains, plasmids and primers used in this study are listed in Table 1.

**Table 1:** Strains, plasmids and oligonucleotides used in this study.

**Strain or plasmid**	**Description**	**Reference or source**
*C. necator* strains		
H16	Wild-type (wt), DSM 428 (*Cupriavidus necator*)	DSMZ
H16::* gfp*	Recombinant strain containing *gfp* fusion vector, derivative of H16	This study
*E. coli* strains		
S17-1	Strain (ATCC 47055) for conjugative transfer of vectors to *C. necator,* recA pro hsdR, RP4-Tc::Mu-Km::Tn7 integrated into the chromosome, tmpR, spcR, strR	ATCC, (Simon, Priefer & Puhler, 1983)
JM109	High Efficiency Competent Cells (>10^8^cfu/μg) for transformation	Promega
Vectors		
pGEM^®^-T Easy	PCR TA cloning vector, ampR	Promega
pGEM-SH*::gfp*	Derivative of pGEM^®^-T Easy containing P_SH_*::gfp* fusion elements	This study
pGLO	Vector carrying the *gfp* gene	Bio-Rad
pJQ200mp18	Suicide vector in *E. coli* DH10B, ATCC 77485. gtmR – EcoRI/MCS/HindIII/PstI – P15A – traJ – oriT – sacB	ATCC, (Quandt & Hynes, 1993)
pJQ200mp18-SH::*gfp*	Derivative of pJQ200mp18 containing P_SH_*::gfp* fusion elements	This study

**Oligonucleotides**	**Sequence**	**Restriction site**
Cloning		
*F-upstream*	CTGCAGCTGCCTCCGGTCACCCGG	PstI
*R-upstream*	GTTGTCTCCTCCTTACTAATGTTCGCC 5′phosphorylated	
*F-gfp*	ATGGCTAGCAAAGGAGAAGAACT 5′phosphorylated	–
*R-gfp*	CTGCAGTTGTCAAATTTTTTCGCGATGCG TGCAGGTATGGCCAGGCACATGT TCTACCCTCTCGTCATTTGTAGAGCTCATCCATGC	PstI
*R-gfp-truncated*	CTGCAGTTGTCAAATTTTTTCGCGATG	PstI
Confirmation		
pUC/M13 Sequencing Forward Primer (Promega)	GTTTTCCCAGTCACGAC	–
pUC/M13 Sequencing Reverse Primer (Promega)	CAGGAAACAGCTATGAC	–
*R-recombination*	CAGGTCGATGAGGGCCATGTCG	–
RT-qPCR		
*hoxF_fwd*	CTGTTCGACACCCCCTGTAT	
*hoxF_rev*	ATAGGCGATGTCCTGACTGG	
*gfp_fwd*	AGTGGAGAGGGTGAAGGTGA	
*gfp_rev*	ACGGGAAAAGCATTGAACAC	
*gyrB_fwd*	GCCTGCACCACCTTGTCTTC	
*gyrB_rev*	TGTGGATGGTGACCTGGATCT	

### DNA isolation, manipulation and amplification

The UltraClean^®^ Microbial DNA Isolation Kit (MO BIO Laboratories, Carlsbad, CA, USA) was used for genomic DNA preparation from *C. necator* H16. After separation of PCR amplified products by agarose gel electrophoresis, DNA fragments were excised from the gel and extracted using the Wizard SV Gel and PCR Clean-up system kit (Promega, Sunnyvale, CA, USA) following the manufacturer’s protocol. The same kit was also used for clean-up of the pJQ200mp18 vector following the restriction enzyme digestion. The Wizard^®^ Plus SV Minipreps DNA Purification System (Promega, Sunnyvale, CA, USA) was used to isolate plasmids from microorganisms according to the manufacturer’s instruction. For DNA amplification, 2X PCR Master Mix (Promega, Sunnyvale, CA, USA) was used. For proof-reading PCR, Phusion™ High-Fidelity DNA Polymerase (Finnzymes, Espoo, Finland) was used with 5x Phusion HF Buffer supplied. The cycling conditions vary depending on the purpose. The ABI 3730 Capillary Sequencer with BigDye™ Terminator Cycle Sequencing Ready Reaction kit v.3.1 (Applied Biosystems) was used for sequencing PCR of cloned insert DNA according to the manufacturer’s instructions.

### Hydrogenase activity assay

Soluble hydrogenase assays were performed as described previously in 50 mM H_2_-saturated Tris/HCl buffer at pH 8.0 (Jugder et al., 2015; Jugder et al., 2016). NAD^+^ was used as an artificial electron acceptor and its reduction to NADH was measured spectrophotometrically at 340 nm.

### Construction of a P_SH_ promoter-*gfp* fusion element

**Figure 1:**
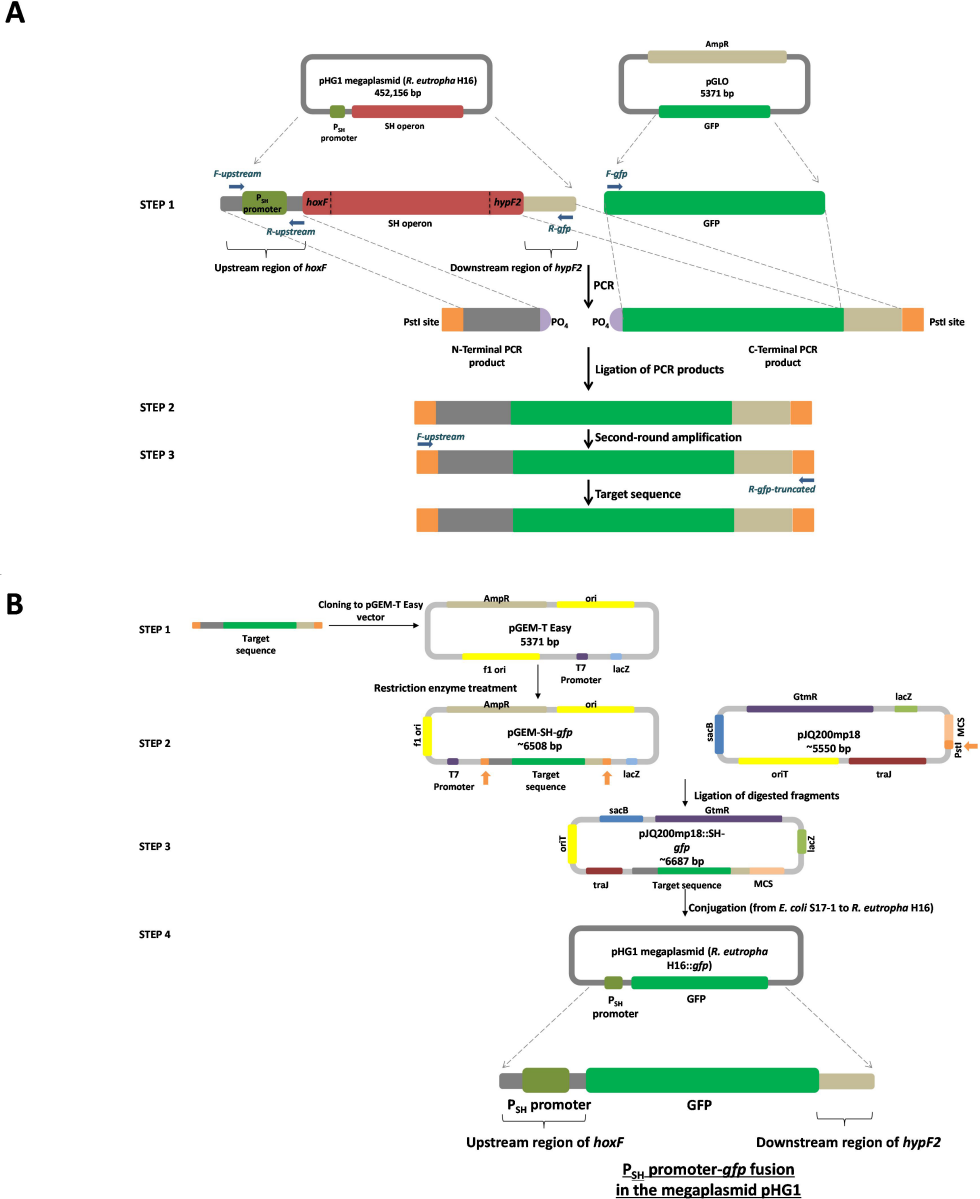
Overview of the molecular cloning method employed in this study. (A) Flow diagram of the steps involved in the generation of the target sequence to be fused. Step 1: the PCR amplification of the region upstream of *hoxF* up to the translational stop codon of the previous ORF (phosphorylated at the 5′ end of the non-coding strand) by using primers *F*-upstream and *R*-upstream and template *C. necator* H16 DNA and a *gfp* plus the portion of the region post *hypF2* amplicon (phosphorylated at the 5′ end of the coding strand) by using primers *F*-*gfp* and *R*-*gfp* and template pGLO. Step 2: ligation of PCR products. Step 3: secondary amplification of the ligated product to generate the target DNA. (B) Flow diagram of the steps involved in the construction of a P_**SH**_ promoter-*gfp* fusion system. Step 1: cloning of the target sequence to pGEM-T Easy vector to generate pGEM-SH*::gfp* vector. Step 2: restriction enzyme digestion of pGEM-SH*::gfp* vector and pJQ200mp18 vectors at the PstI endonuclease site (shown by the orange arrows). Step 3: ligation of the digested target sequence to the digested pJQ200mp18 vector to generate pJQ200mp18-SH*::gfp* vector. Step 4. conjugation of the recombinant vector pJQ200mp18-SH*::gfp* from *E. coli* S17-1 to *C. necator* H16 to construct the integrated final P_SH_ promoter-*gfp* fusion system.

An overview of the creation of the P_SH_ promoter-*gfp* fusion elements is shown in Fig. 1. A 353 bp fragment, phosphorylated at the 5′ end, containing the region upstream of *hoxF* (nucleotides 79,365 and 79,382, sequence numbering according to GenBank entry AY305378.1) up to the translational stop codon of the previous ORF (nucleotides 79,685 and 79,711, sequence numbering according to GenBank entry AY305378.1) was amplified in the first PCR by using primers *F-upstream* and *R-upstream*, and *C. necator* H16 chromosomal DNA as template. Similarly, a 784 bp fragment, containing a *gfp* plus the portion of the region post *hypF2* amplicon (nucleotides 89,228 and 89,285, sequence numbering according to GenBank entry AY305378.1) and phosphorylated at the beginning, was generated from pGLO template, which harbours the *gfp* gene, by using primers *F-gfp* and *R-gfp*. Thus, all transcriptional control and stop elements of the SH are located in the regions amplified by these primer pairs. These fragments were, after gel-extraction, ligated using T4 DNA Ligase (Promega) and subjected to a further PCR by using primers *F-upstream* and *R-gfp-truncated* to amplify the ligation product of the expected size of 1,137 bp (Fig. S1). The gel-purified ligated fragment was sub-cloned into the pGEM-T Easy vector and the resultant recombinant vector was designated as pGEM-SH*::gfp* (Fig. 1B), which was used for transformation of *E. coli* JM109. The transformation culture was plated in duplicate on LB/ampicillin/IPTG/X-Gal plates for further blue-white screening. Sequencing PCR was performed on purified pGEM-SH*::gfp* vectors from white colonies employing the pUC/M13 Sequencing Forward and Reverse Primers.

The pJQ200mp18 suicide vector was used in this work to carry out gene replacement and mobilization experiments (Quandt & Hynes, 1993). Mini-preps of the pJQ200mp18 suicide vector and pGEM-SH*::gfp* vector DNA were digested with PstI restriction endonuclease and dephosphorylated using Antarctic Phosphatase for further ligation to yield the recombinant vector pJQ200mp18-SH*::gfp*, which was used to transform *E. coli* JM109. Following blue/white screening (LB/gentamicin /IPTG/X-Gal selective plates), the recombinant vector purified from *E. coli* JM109 was used to transform *E. coli* S17-1 competent cells via a heat shock at 42°C for 1 min. The transformed competent cells were plated onto LB/gentamicin/trimethoprim/IPTG/X-Gal plates, as *E. coli* S17-1 harbouring pJQ200mp18 is resistant to gentamicin (Quandt & Hynes, 1993) and trimethoprim (Simon, Priefer & Puhler, 1983). The mobilisable suicide vector, pJQ200mp18-SH*::gfp*, was transferred from *E. coli* S17-1 to *C. necator* H16 by spot mating (Dernedde et al., 1996). Transconjugants were selected by plating serial dilutions on low-salt LB plates containing 5% sucrose. After 3–5 days of incubation at 30 °C, transconjugants appeared and colony PCR was used to screen transconjugants, with primers *F-gfp* and *R-recombination*. Transconjugants were inoculated into 5 mL of FGN media and incubated overnight at 30°C. Genomic DNA from select transconjugants was subjected to final PCR using the primers *F-gfp* and *R-recombination*, and primers *F-upstream* and *R-gfp-truncated* to amplify DNA fragments of approximate 800 bps and 1.14 kbps, respectively, in order to confirm final successful recombination.

### Transcriptional analysis

Total RNA extraction and subsequent cDNA synthesis were performed using the TRIzol Plus RNA Purification Kit (Life Technologies, Carlsbad, CA, USA) and the SuperScript III First-Strand Synthesis System (Life Technologies), respectively, as described in Jugder et al. (2015). Expression levels of the *hoxF* gene encoding HoxF protein (NAD-reducing hydrogenase diaphorase moiety large subunit) of the SH in different growth phases of wild-type *C. necator* were analysed using qRT-PCR with primers *hoxF_fwd* and *hoxF_rev*. In the conjugated strains, expression of *gfp* gene was examined with *gfp_fwd* and *gfp_rev* primers. The *gyrB* gene was used as an internal reference gene due to its constitutive expression. qRT-PCR was performed on a Rotor-Gene RG-3000A cycler (Qiagen, Chadstone Centre, VIC, Australia) using the SensiFAST SYBR No-ROX Kit (Bioline, Eveleigh, NSW, Australia) as described elsewhere (Jugder et al., 2015).

### Fluorescence microscopy examination of the transconjugants to detect the presence of GFP

The cultures that were inoculated from single colonies from the conjugated strains were grown overnight in 5 mL GN (Glycerol as sole carbon source, hydrogenase de-repressing condition) and FN media (Fructose as sole carbon source, hydrogenase repressing condition). The overnight cultures were placed on glass slides with cover slips and examined for brightfield imaging under light microscope settings with 10*x* and 50*x* objectives for locating the cells. Subsequently, the cells were examined for fluorescence by using the “WB” filter tube, which is a combination of a BP450-480 excitation filter, a DM500 dichroic mirror and a BA515 barrier filter (filter cube WB). This combination elicited a green fluorescence of the transconjugants expressing GFP. The images of the GFP-expressing cells under fluorescence settings were obtained using DP Manager v3.3.1.222 software (Olympus).

### Flow cytometry analysis of GFP

The cultures that were inoculated from single colonies from the conjugated strains were grown overnight in 5 mL GN (hydrogenase de-repressing) and FN (hydrogenase repressing) media. After two successive 400-fold dilutions, 5×10^4^ cells from each pool were analyzed using a Becton-Dickinson FACS Caliber flow cytometer, and fluorescence (488-nm excitation, 520-nm emission) was scaled by scattering to compensate for differences in cell morphology and size. One hundred thousand events (cells) were counted for each sample. Experiments were performed in triplicate unless otherwise stated.

### Purification of GFP isolated from transformed *C. necator*

The cell pellets were harvested by centrifugation at 5,500 g for 15 min at 5 °C, and stored at −80 °C. Cells were disrupted by sonication and the cell-free extract was centrifuged (100,000 g, 30 min at 5 °C). The remaining supernatant was loaded onto a 10-ml volume metal affinity resin (Talon resin; Clontech) equilibrated in buffer containing 150 mM NaCl, 100 mM HEPES–NaOH, pH 7.5. Unbound proteins were washed off using the same buffer containing 10 mM imidazole. The bound protein was then eluted with a buffered solution composed of 200 mM imidazole, 150 mM NaCl, 100 mM HEPES-NaOH, pH 7.5. The solution containing the precipitated protein was centrifuged, and the supernatant was discarded. The precipitate was progressively dissolved in 20 mM HEPES-NaOH, pH 7.5. The protein solution was dialyzed overnight against a 500-fold (vol/vol) excess of the same solution.

### Absorption and fluorescence excitation and emission spectra

Samples of purified GFP were diluted to approximately 4.5 µM in buffered solution (containing 10 mM glycine, 10 mM sodium citrate, 10 mM sodium phosphate, and 5 mM Tris-HCl). A fluorometer (Fluorostar Optima) was used to obtain the emission spectrum of the commercial GFP and the GFP extracted and purified from the transconjugant *C. necator* H16. Measurements were obtained using excitation and emission wavelengths, bandpass, and integration times of 392 nm, 510 nm, 3 nm, and 0.5 s, respectively.

### Fluorescence quantitation in wildtype and transformed *C. necator*

The fluorescence intensity of GFP in fixed cells was measured with a Fluoromax-2 spectrofluorometer using the Datamax for Windows software interface (Instruments S.A. Inc., Edison, NJ, USA). A protein assay on lysates of the cell samples was carried out prior to normalise cell loading for gfp fluorescence determination, using the Pierce BCA Protein Assay Kit (Thermo Scientific, Waltham, MA, USA). The relative fluorescence unit (RFU) is defined as the culture fluorescence relative to culture concentration (OD_600*nm*_).

## Results and Discussion

In this study, the transcriptional reporter method was employed to construct the P_SH_ promoter-*gfp* fusion in the megaplasmid pHG1 of *C. necator* H16 to analyse promoter activity. The molecular cloning method was designed to generate, by PCR, the entire sequence of the 5′ upstream elements which were subsequently fused to the *gfp* gene that was combined with 3′ downstream elements of the SH operon by establishing a rapid and robust cloning approach which is summarised in Fig. 1. The *gfp* gene from a commercially available pGLO vector was fused to the P_SH_ promoter of the SH operon in place of the first ORF (*hoxF*) followed by 3′ downstream elements following the final ORF (*hypF2*) of the same operon. The results confirmed that the fusion elements recombined with the pHG1 megaplasmid of wild-type *C. necator* by a means of gene replacement at the site of the SH operon elements. The resulting reporter construct was capable of being induced under the hydrogenase de-repressing condition (GN medium) in the transconjugant derivative cells which led to detectable fluorescence signals from the GFP expressed.

Initially, the 784 bp amplicon representing a GFP product combined with the region downstream of *hypF2* (using the primers *F-gfp* and *R-gfp* as well as pGLO vector) and the 353 bp amplicon from the region upstream of *hoxF* (using the primers *F-upstream* and *R-upstream*, and *C. necator* H16 chromosomal DNA as template) were obtained (Fig. S2A). The ligation reaction of these two fragments theoretically can result in three possible ligated products joined via the 5′-phosphorylated ends (Fig. S2B) as follows: (i) between two *N*-terminal products, (ii) between an *N*-terminal product and a *C*-terminal product and (iii) between two *C*-terminal products. The second product is the desired ligation product with a calculated size of 1,137 bp which was excised from a gel for further PCR amplification by using the primers *F-upstream* and *R-gfp-truncated* (Fig. S2B). In Fig. S3, gel images of the results of each cloning step are shown: the colony PCR product with the expected theoretical size (1,137 bp) generated from a JM109 transformant harbouring the pGEM-SH*::gfp* vector (Fig. S3A), the PstI-digested fragments of the isolated pGEM-SH*::gfp* vector and the pJQ200mp18 suicide vector prior to the ligation to yield the vector pJQ200mp18-SH*::gfp* (Fig. S3B) and confirmation of successful sub-cloning of the resulting recombinant suicide vector by PstI digestion (Fig. S3C). The colony PCR product from *E. coli* S17-1 transformed with the pJQ200mp18-SH*::gfp* vector using the primers *F-upstream* and *R-gfp-truncated* enabled rapid screening for successful transformation (Fig. S3D). Furthermore, the colony PCR product with the estimated size of 800 bp from a transconjugant *C. necator* H16*::gfp* cell after spot-mating by using primers *F-gfp* and *R-recombination* (Fig. S3E) on a gel indicated successful gene replacement on the megaplasmid pHG1. These transconjugants were designated as *C. necator* H16*::gfp* cells. Lastly, PCR was performed using two primer pairs (*F-upstream* and *R-gfp-truncated*, as well as *F-gfp* and* R-recombination*) on genomic DNA prepared from the transconjugant colonies which confirmed successful transformation (Fig. S3F).

**Figure 2:**
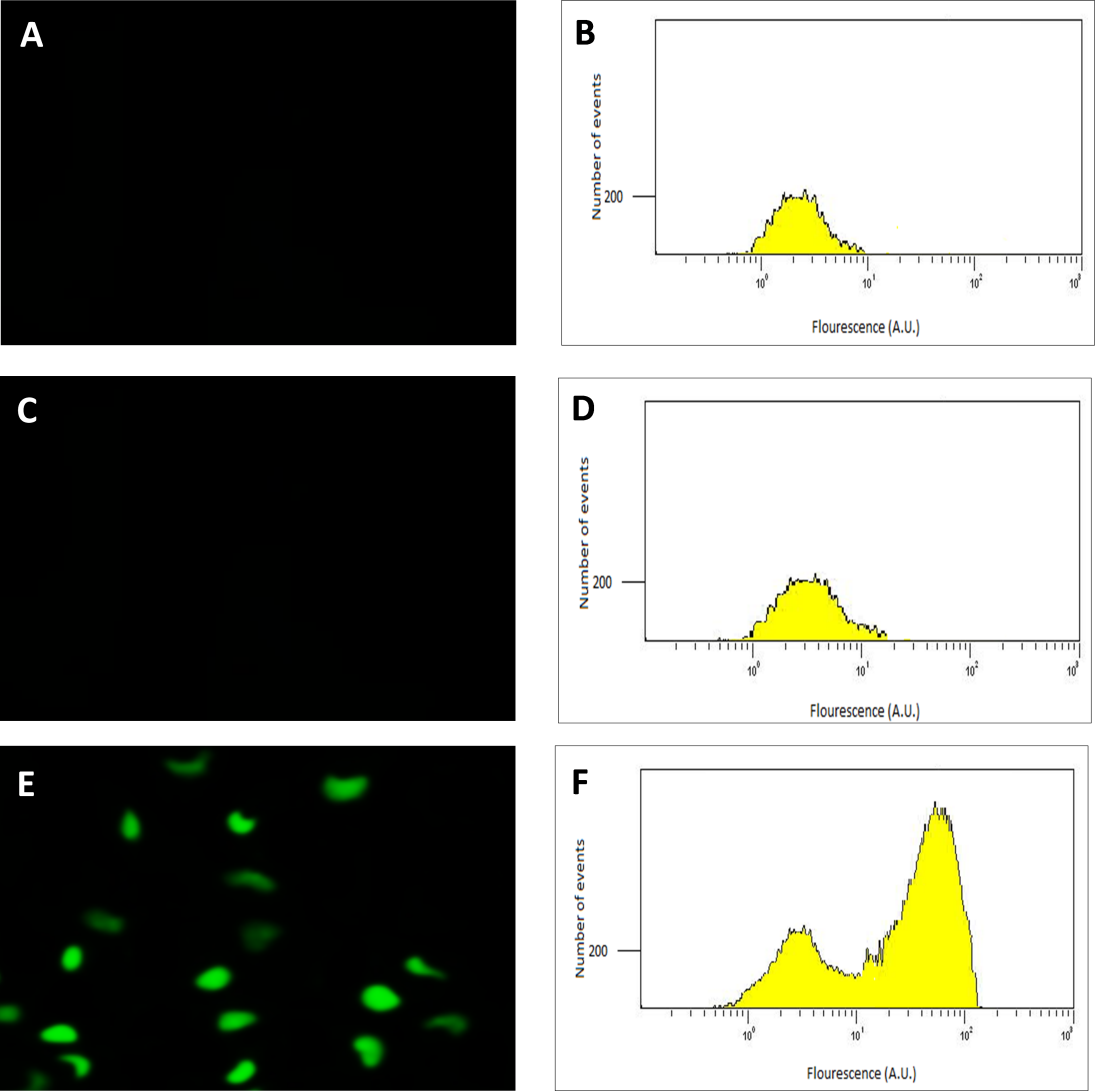
Detection of GFP-expressing *C. necator* H16*::gfp* cells. The fluorescence images (A, C and E) of the cells with corresponding flow cytometry fluorescence histograms (B, D and F). The GFP signal was not detected from wild-type *C. necator* H16 cells (A and B) and the transformed *C. necator* H16*::gfp* cells under the hydrogenase repressing condition (growth on fructose) (C and D), whereas the GFP signal was detectable in transformed *C. necator* H16*::gfp* cells under the hydrogenase de-repressing condition (growth on glycerol) (E and F).

Following the confirmation of the successful final recombination event, the performance of the transconjugant (*C. necator* H16*::gfp*), in producing GFP under control of the P_SH_ promoter, was determined using fluorescence microscopy. Glycerol stocks were subsequently prepared from cultures derived from single colonies that demonstrated green fluorescence when grown in GN media. Quantitative RT-PCR, flow cytometry and quantitative fluorescence analysis was subsequently undertaken using cultures from these glycerol stocks. Images and flow cytometry data of the cells expressing GFP under the hydrogenase repressing condition (fructose; FN media) and the hydrogenase de-repressing condition (glycerol; GN media) were obtained (Fig. 2). GFP expression was observed visually and by a significant shift in the population, verifying that the P_SH_ promoter from the transconjugated *C. necator* successfully induced GFP production under the selected hydrogenase de-repressing growth condition.

**Figure 3:**
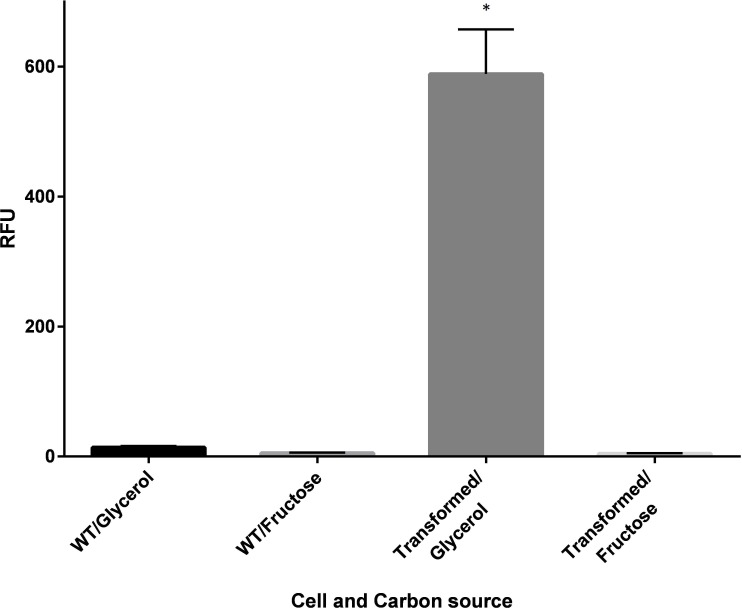
Fluorescence of wildtype (WT) and recombinant *C. necator* (transformed) in fructose (FN) media and glycerol (GN) media. Specific fluorescence response (RFU) of *C. necator* H16*::gfp* (transformed) and non-transformed (WT- wild-type) cells excited at 392 nm under repressing conditions (growth on fructose) and de-repressing conditions (growth on glycerol). Bars represent the mean ± S.E gfp relative fluorescence units obtained from triplicates for each treatment group. Significance ^∗^*p* < 0.01 compared to wild-type *C. necator* H16 cells in glycerol and fructose, and the transformed *C. necator* H16*::gfp* cells under the hydrogenase repressing condition (fructose). *P*-values were calculated using one way analysis of variance (ANOVA) followed by a *t*-test.

The emission characteristics of the recombinant GFP isolated from the transconjugants confirmed its authenticity, with emission maxima observed at excitation wavelengths of 392 and 475 nm (Fig. S4) coinciding exactly with that of the native GFP. In the fluorescence plate assay, a significant increase in GFP expression was demonstrated under P_SH_ de-repressing conditions (growth in GN media) for the transformed population (Fig. 3).

**Figure 4:**
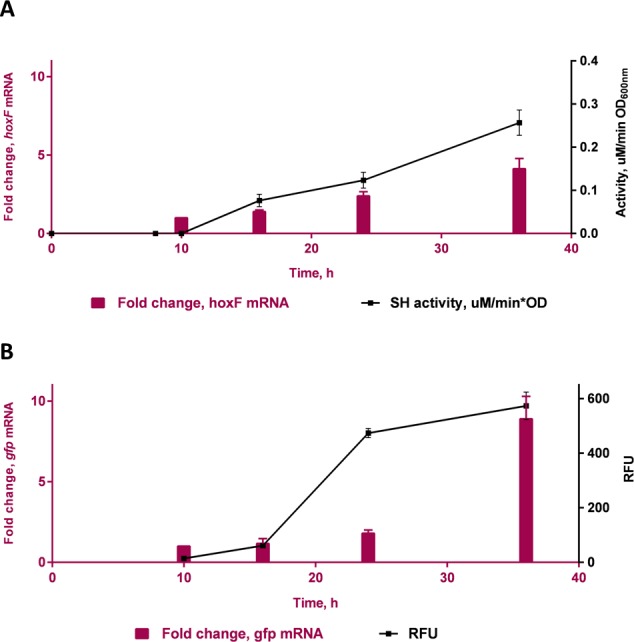
Transcriptional analyses of SH operons. Differential expression of (A) *hoxF* gene (*P* value 0.0039) and NAD^+^ reducing soluble hydrogenase (SH) activity (*P* value 0.0012) from wild-type *C. necator* H16 cells and (B) *gfp* gene (*P* value 0.0493) and GFP (*P* value 0.0303) in *C. necator* H16*::gfp* cells, respectively. These graphs are based on three technical replicates and represent their mean values with standard deviation indicated by the error bars. Constructed and analysed by GraphPad Prism, v 6.07. *P*-values were calculated using one way analysis of variance (ANOVA) followed by a *t*-test.

A time course study in FGN media (Fig. 4) showed increasing protein expression (soluble hydrogenase in the WT strain and GFP fluorescence in the transformed strain) and increased fold change in respective mRNA levels, as cells switched from growth on fructose (*t* = 10 h) to growth on glycerol (*t* = 16 h, 24 h and 36 h). The gene *hoxF* was approximately 1.4, 2.1 and 3.5-fold up-regulated in the cells harvested at 16 h, 24 h and 36 h where the expression of SH was assumed to be induced, in comparison to the cells at 10 h (Fig. 4A). The SH expression was also demonstrated as specific SH activity increased in accordance with the increase in abundance of *hoxF* mRNA. In parallel, the *gfp* gene expression was investigated in the conjugated cells at the transcriptional level (Fig. 4B). We observed the up-regulation of the *gfp* gene with an approximate 8.9-fold increase at 36 h. Observations made in the time course of the expression pattern of the genes *hoxF* and *gfp* confirmed that P_SH_ promoter, in our constructed strain, is responsive to the de-repression upon carbon source change in a similar manner. Together, these findings confirm the utility of the transformed *C. necator* H16*::gfp* for future P_SH_ activity screening.

To our knowledge, this is the first report of a successful fluorescent reporter system to study P_SH_ promoter activity in *C. necator* H16. Understanding the environmental factors in the regulation of SH expression is of increasing interest and the availability of versatile monitoring methods is crucial. The system developed in this study should allow for the conduct of factorial experiments and high-throughput assays in a microplate format that employs the recombinant *C. necator* H16*::gfp* cells to explore alternative growth conditions and rapidly estimate SH promoter activity. Furthermore, there is potential to use this construct in transposon mutagenesis experiments to identify new SH regulators by monitoring a simple fluorescence read-out. This tool has the potential to further assist in investigating the sigma factor, *σ*^54^, which recognises the P_SH_ promoter (Schwartz, Gerischer & Friedrich, 1998). Possible carbon sources could theoretically be identified as ideal candidates to induce strongly the *σ*^54^-dependent P_SH_ promoter. Also, evaluation of site-directed mutagenesis of the P_SH_ promoter or the replacement of the P_SH_ promoter with a more strongly inducible promoter could be facilitated by this reporter system. The generation of these reporter strains is based on recombination events; further characterization of a range of the recombinant transconjugants may also reveal as yet unidentified variants that possess useful traits that may assist in the identification of inducing conditions.

## Conclusion

In the present study, a system to investigate soluble hydrogenase P_SH_ promoter activity in *C. necator* H16 was constructed and its functionality was confirmed, developing a P_SH_-GFP fusion protein reporter. A series of molecular cloning steps were employed to replace the ORF of the SH with a *gfp* gene in the megaplasmid pHG1, and the expression of GFP in response to the de-repression of the SH genes was demonstrated under fluorescence and transcriptional analyses. This construct will enable future studies to design simple screening methods for P_SH_ promoter activity in *C. necator* H16 cells, further investigations on growth-related optimisation with alternative cultivation conditions and functionality of P_SH_ promoter mutants in *C. necator*.

##  Supplemental Information

10.7717/peerj.2269/supp-1 Figure S1 The target sequence generated by primers *F-upstream* and *R-upstream, F-gfp* and *R-gfp* (or *R-gfp-truncated*) Keys: Lower case only: corresponds to a region upstream of *hoxF* up to the translational stop codon of the previous ORF (encodes putative transposase). All transcriptional control elements are located within this region. Caps: *gfp* sequence from a pGLO vector but translational stop changed to TGA to correspond with the most common translational stop (UGA) found in *C. necator*. Lower case italics: a portion of the region post *hypF2* that includes transcriptional stops. Underlined are the PstI restriction sites.

10.7717/peerj.2269/supp-2 Figure S2 Amplifications and ligations of the SH operon elements and the *gfp* gene (A) 1.5% agarose gel of PCR amplicons from transcriptional control and stop elements of the soluble hydrogenase operon of *C. necator*H16 and a *gfp* gene in pGLO vector (primary amplification). Lane 1: PCR product from pGLO template generated by primers *F-gfp* and *R-gfp* (784 bp), Lane 2: 100bp DNA Ladder (Promega), Lane 3: PCR product from *C. necator*H16 chromosomal DNA template generated by *F-upstream* and *R-upstream* primers (353 bp). (B) Ligation of primary PCR amplicons and secondary amplification. On the left side is 1% agarose gel of the amplified desired ligation product (secondary amplification). Lane 1: 1 kb DNA Ladder (Promega), Lane 2: An expected 1137 bp PCR product from the ligated DNA fragments (template) and using the *F-upstream* and *R-gfp-truncated* primers (relevant band indicated within the red box). On the right side depicted are possible ligations between primary PCR amplicons. A N-Terminal PCR product contains an upstream region of *hoxF* (grey), whereas a C-Terminal product contains a *gfp* sequence (green) followed by a downstream region of *hypF2* (tan)*.* Phosphorylated ends are shown in purple and PstI sites are in orange. Three possible ligations are i) between two N-terminal products, ii) between an N-terminal product and a C-terminal product (the target insert DNA) and iii) between two C-terminal products.

10.7717/peerj.2269/supp-3 Figure S3 Agarose gel visualizations of gene products of cloning steps (A) 1% agarose gel of the colony PCR product generated from a JM109 transformant harbouring the pGEM-SH*::gfp* vector. Lane 1: 1 kb DNA Ladder, Lane 2: a 1137 bp PCR product generated from a white colony after transformation (within the red box). (B) 1% agarose gel of the digested fragments. Lane 1: 1 kb DNA Ladder, Lane 2: The PstI-digested pGEM-SH*::gfp* vector is separated into an approximately 3 kb pGEM-T Easy vector and a 1.1 kb insert fragment of SH operon elements fused to *gfp* (within the red box), Lane 3: The PstI-digested pJQ200mp18 vector of 5.5 kb (within the blue box) and Lane 4: undigested pGEM-SH*::gfp* vector. (C) 1% agarose gel of the digested pJQ200mp18-SH*::gfp* vector. Lane 1: 1 kb DNA Ladder, Lane 2: the 1137 bp insert fragment released from the PstI-digested pJQ200mp18-SH*::gfp* vector isolated from a white colony after transformation (within the red box). (D) 1% agarose gel of the colony PCR product generated from a transformant harbouring the pJQ200mp18-SH*::gfp* vector in *E. coli* S17-1 cells. Lane 1: 1 kb DNA Ladder, Lane 2: the 1137 bp PCR product generated from a white colony after transformation (within the red box). (E) 1% agarose gel of the colony PCR product generated from a transconjugant *C. necator* H16*::gfp* cell. Lane 1: the 800 bp PCR product generated from a transconjugant colony after conjugation (within the red box), Lane 2: 1 kb DNA Ladder. (F) 1% agarose gel of amplicons generated from *C. necator* H16*::gfp* cells. Lane 1: the 800 bp PCR product generated from a transconjugant with primers *F-gfp* and* R-recombination* (within the red box), Lane 2: the 1.14 kb PCR product generated from a transconjugant with primers *F-upstream* and *R-gfp-truncated* (within the blue box), Lane 3: 1 kb DNA Ladder.

10.7717/peerj.2269/supp-4 Figure S4 Fluorescence of purified and cellular recombinant GFP Emission spectrum of extracted protein (507 nm), at different excitation wavelengths, with maxima observed at 392 and 475 nm, is shown to coincide with that of native GFP.

## Additional Information and Declarations

### Data Availability

The following information was supplied regarding data availability: Sequence information has been supplied as Supplementary files.
