# Supplementary figures and images for: Construction and use of a Cupriavidus necator H16 soluble hydrogenase promoter (PSH) fusion to gfp (green fluorescent protein)
Source: PeerJ. 2016 Jul 26;4:e2269. doi: 10.7717/peerj.2269 (PMC4974937; doi:10.7717/peerj.2269)

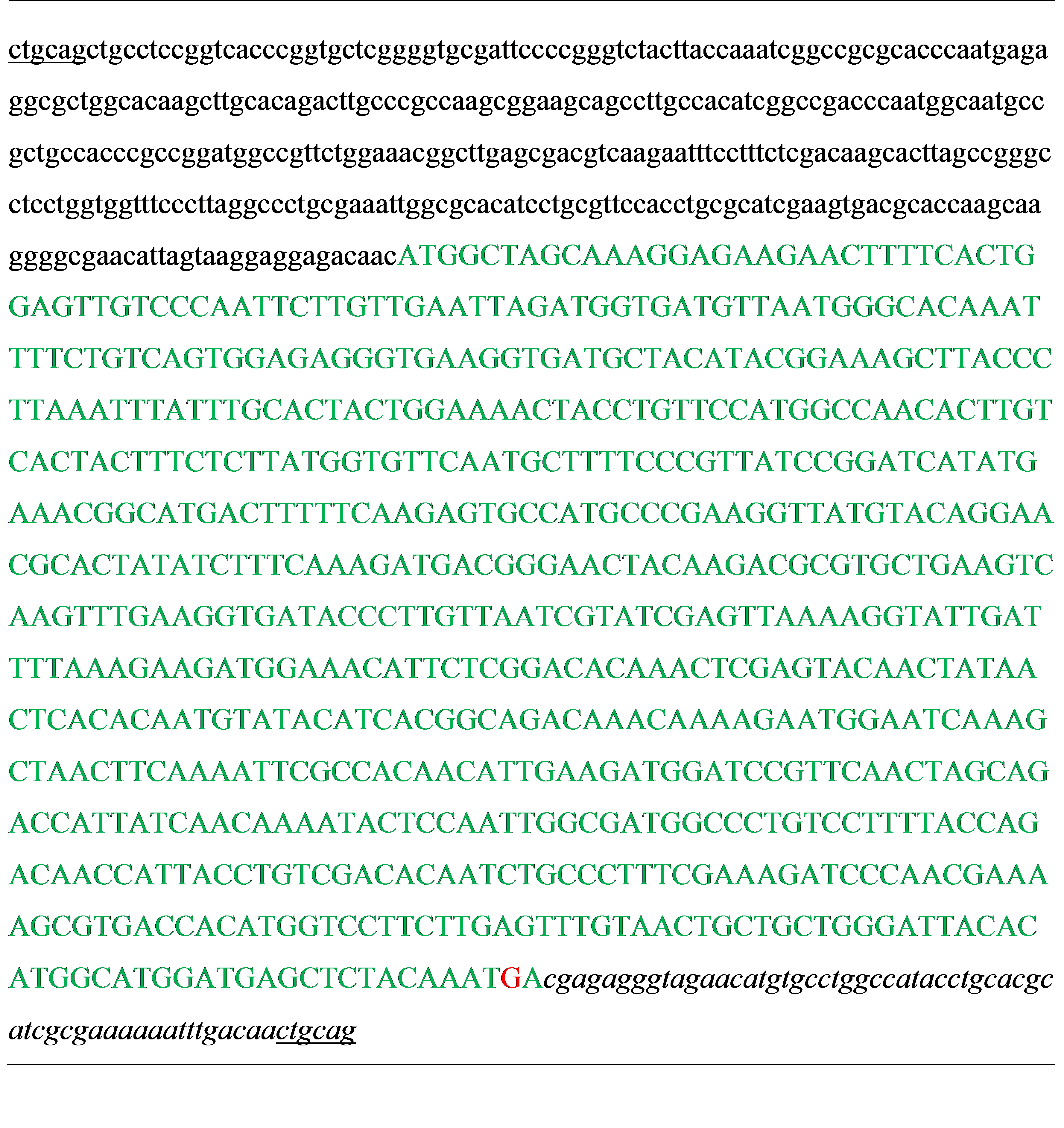

Supplement: Figure S1 — Keys: Lower case only: corresponds to a region upstream of hoxF up to the translational stop codon of the previous ORF (encodes putative transposase). All transcriptional control elements are located within this region. Caps: gfp sequence from a pGLO vector but translational stop changed to TGA to correspond with the most common translational stop (UGA) found in C. necator. Lower case italics: a portion of the region post hypF2 that includes transcriptional stops. Underlined are the PstI restriction sites. [file peerj-04-2269-s001.tif]

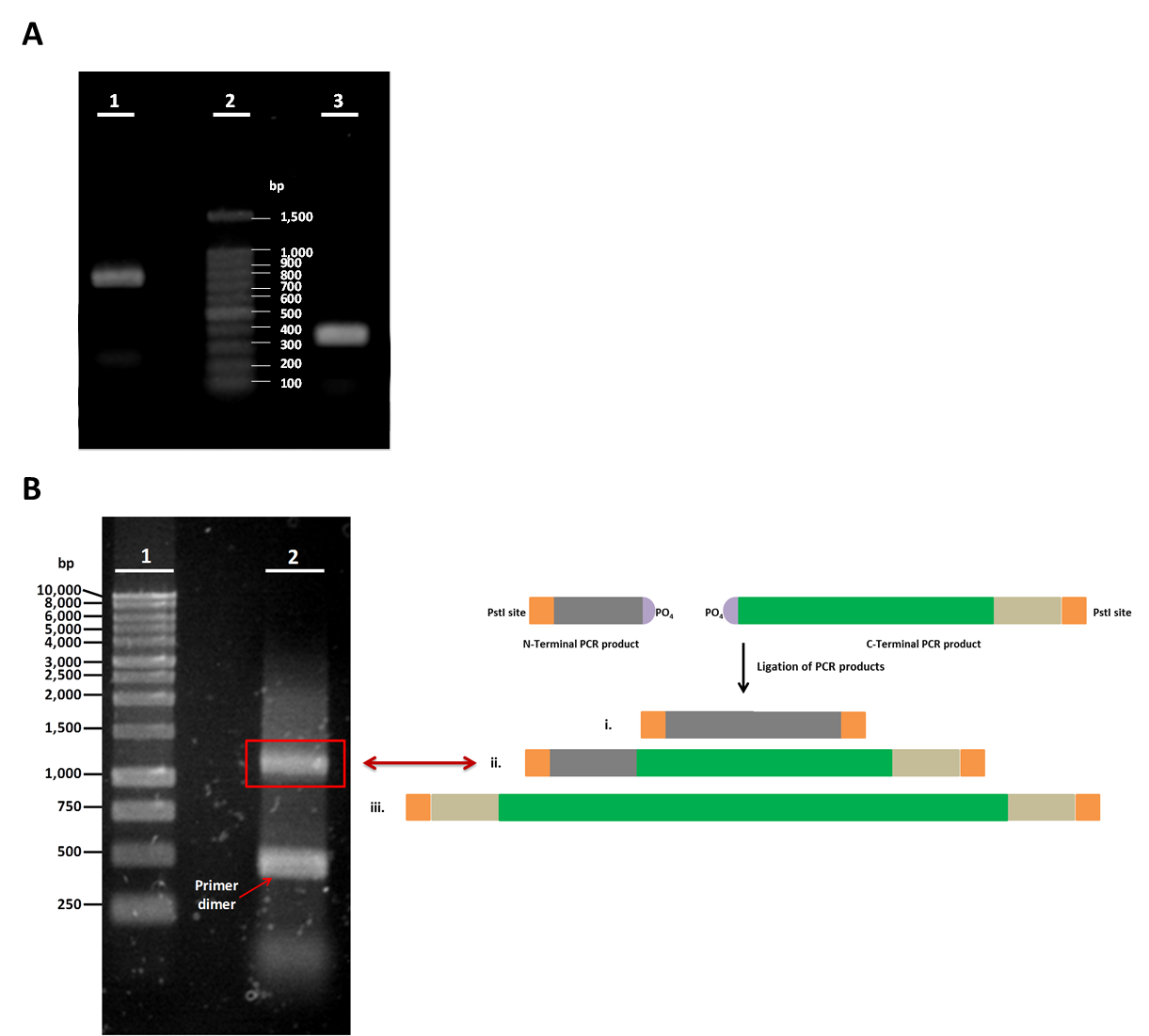

Supplement: Figure S2 — (A) 1.5% agarose gel of PCR amplicons from transcriptional control and stop elements of the soluble hydrogenase operon of C. necatorH16 and a gfp gene in pGLO vector (primary amplification). Lane 1: PCR product from pGLO template generated by primers F-gfp and R-gfp (784 bp), Lane 2: 100bp DNA Ladder (Promega), Lane 3: PCR product from C. necatorH16 chromosomal DNA template generated by F-upstream and R-upstream primers (353 bp). (B) Ligation of primary PCR amplicons and secondary amplification. On the left side is 1% agarose gel of the amplified desired ligation product (secondary amplification). Lane 1: 1 kb DNA Ladder (Promega), Lane 2: An expected 1137 bp PCR product from the ligated DNA fragments (template) and using the F-upstream and R-gfp-truncated primers (relevant band indicated within the red box). On the right side depicted are possible ligations between primary PCR amplicons. A N-Terminal PCR product contains an upstream region of hoxF (grey), whereas a C-Terminal product contains a gfp sequence (green) followed by a downstream region of hypF2 (tan). Phosphorylated ends are shown in purple and PstI sites are in orange. Three possible ligations are i) between two N-terminal products, ii) between an N-terminal product and a C-terminal product (the target insert DNA) and iii) between two C-terminal products. [file peerj-04-2269-s002.tif]

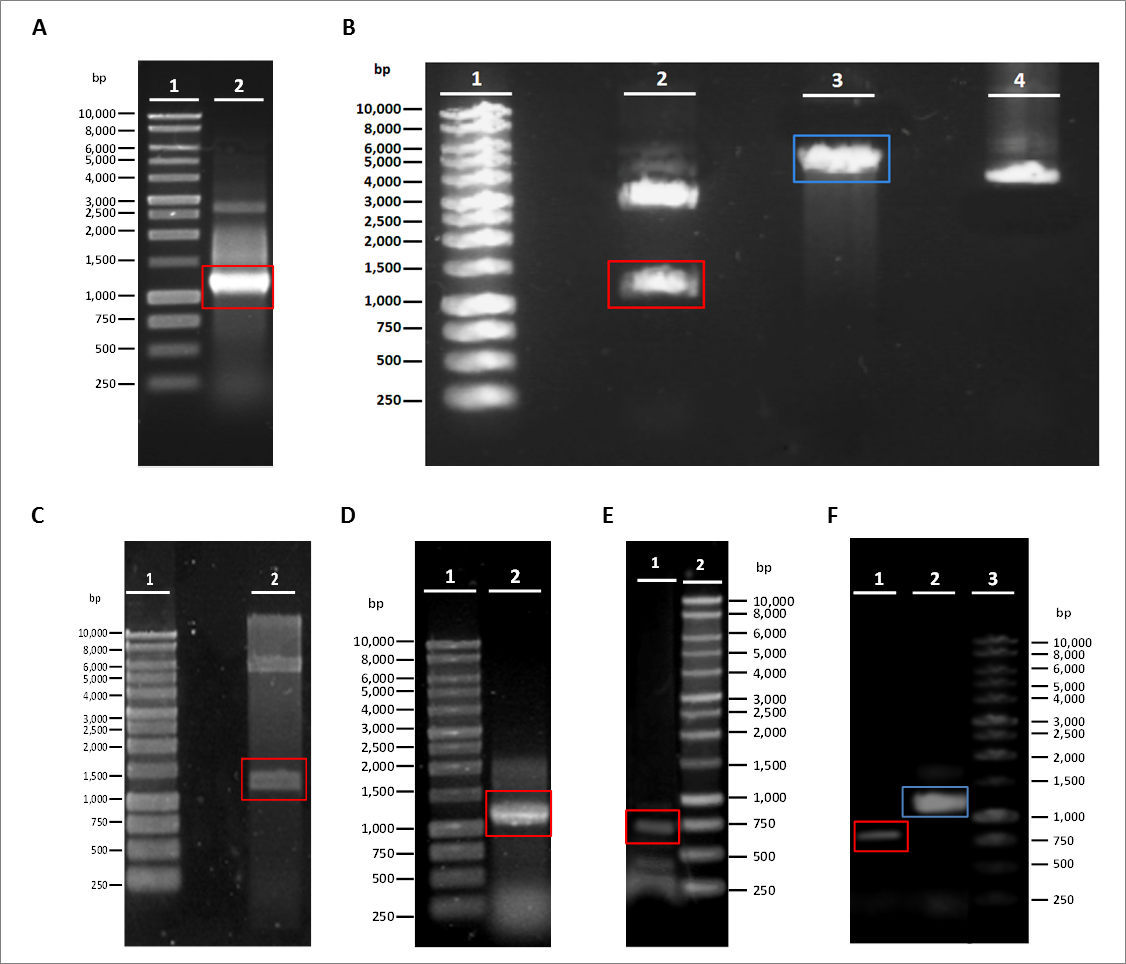

Supplement: Figure S3 — (A) 1% agarose gel of the colony PCR product generated from a JM109 transformant harbouring the pGEM-SH::gfp vector. Lane 1: 1 kb DNA Ladder, Lane 2: a 1137 bp PCR product generated from a white colony after transformation (within the red box). (B) 1% agarose gel of the digested fragments. Lane 1: 1 kb DNA Ladder, Lane 2: The PstI-digested pGEM-SH::gfp vector is separated into an approximately 3 kb pGEM-T Easy vector and a 1.1 kb insert fragment of SH operon elements fused to gfp (within the red box), Lane 3: The PstI-digested pJQ200mp18 vector of 5.5 kb (within the blue box) and Lane 4: undigested pGEM-SH::gfp vector. (C) 1% agarose gel of the digested pJQ200mp18-SH::gfp vector. Lane 1: 1 kb DNA Ladder, Lane 2: the 1137 bp insert fragment released from the PstI-digested pJQ200mp18-SH::gfp vector isolated from a white colony after transformation (within the red box). (D) 1% agarose gel of the colony PCR product generated from a transformant harbouring the pJQ200mp18-SH::gfp vector in E. coli S17-1 cells. Lane 1: 1 kb DNA Ladder, Lane 2: the 1137 bp PCR product generated from a white colony after transformation (within the red box). (E) 1% agarose gel of the colony PCR product generated from a transconjugant C. necator H16::gfp cell. Lane 1: the 800 bp PCR product generated from a transconjugant colony after conjugation (within the red box), Lane 2: 1 kb DNA Ladder. (F) 1% agarose gel of amplicons generated from C. necator H16::gfp cells. Lane 1: the 800 bp PCR product generated from a transconjugant with primers F-gfp and R-recombination (within the red box), Lane 2: the 1.14 kb PCR product generated from a transconjugant with primers F-upstream and R-gfp-truncated (within the blue box), Lane 3: 1 kb DNA Ladder. [file peerj-04-2269-s003.tif]

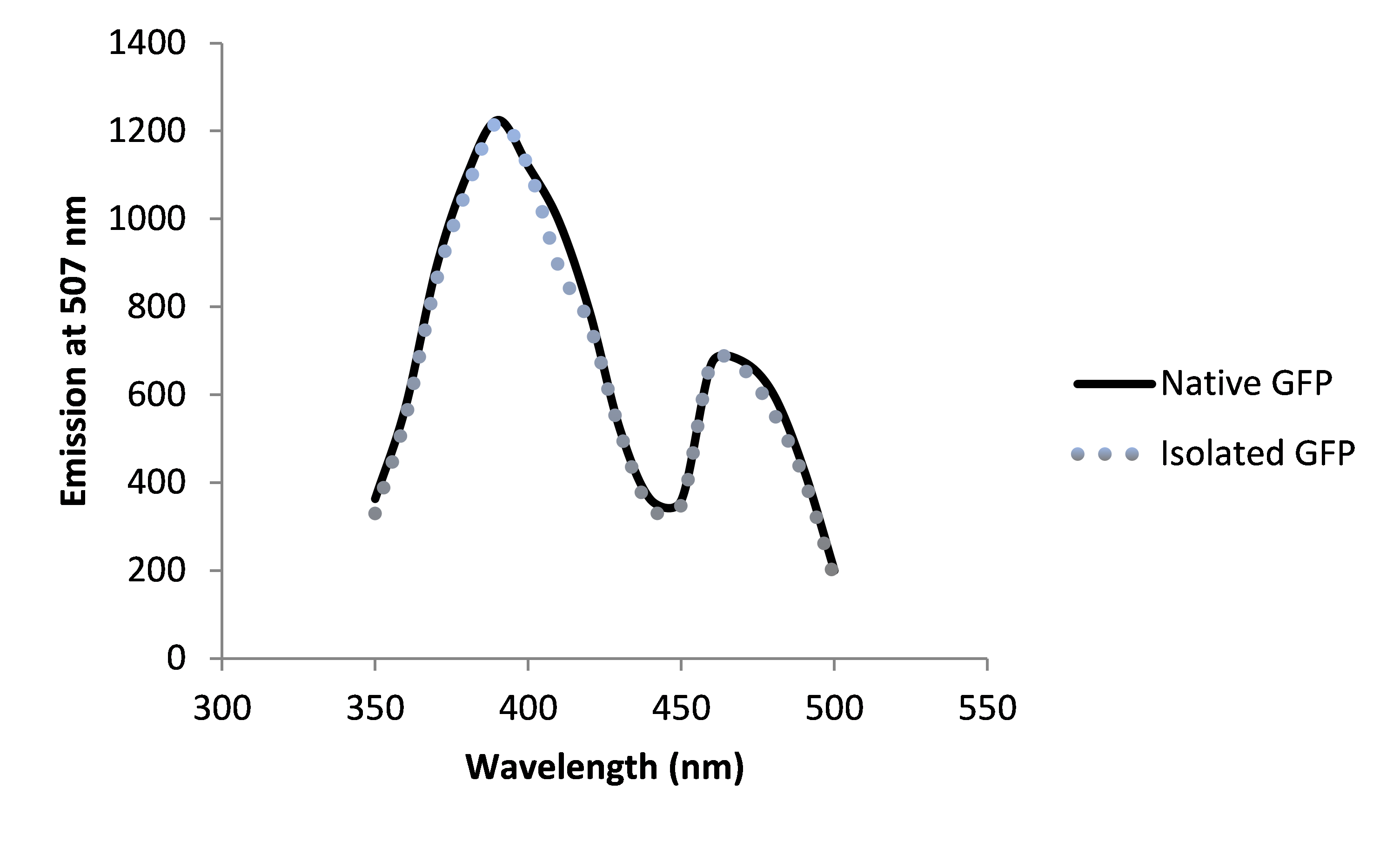

Supplement: Figure S4 — Emission spectrum of extracted protein (507 nm), at different excitation wavelengths, with maxima observed at 392 and 475 nm, is shown to coincide with that of native GFP. [file peerj-04-2269-s004.tif]
